# Intraoperative radiation therapy as part of planned monotherapy for early-stage breast cancer

**DOI:** 10.1007/s13566-017-0338-z

**Published:** 2017-12-19

**Authors:** Varun K. Chowdhry, Julie A. Bushey, Rebecca M. Kwait, Saveli Goldberg, Jeannine Ritchie, Yong-Li Ji, Roderick McKee, Diane Palladino, Gary M. Proulx

**Affiliations:** 1 Department of Radiation Oncology, Massachusetts General Hospital, Boston, MA USA; 2 Department of Radiation Oncology, Exeter Hospital, Exeter, NH, USA; 3 Core Physicians, Department of Surgery, Exeter Hospital, Exeter, NH, USA; 4 Department of Pathology, Exeter Hospital, Exeter, NH, USA; 5 Division of Hematology/Oncology, Massachusetts General Hospital, Boston, MA USA

**Keywords:** Intraoperative radiation therapy, Early-stage breast cancer, Electronic brachytherapy

## Abstract

**Introduction:**

Adjuvant whole breast radiation therapy has developed into the standard of care for patients following a lumpectomy for early-stage breast cancer. However, there is recent interest in intraoperative radiation therapy (IORT) to minimize toxicity while still improving local control beyond surgical resection and anti-estrogen therapy alone.

**Materials and methods:**

All patients were evaluated pre-operatively in a multidisciplinary clinic setting at a community hospital for suitability for breast conservation therapy. A total of 109 patients were reviewed receiving 110 IORT treatments. Patients were followed with clinical breast examinations and mammography as clinically indicated.

**Results:**

At a median follow-up of 29.9 months, 2/110 (1.8%) patients experienced a local failure. One patient (0.9%) experienced a regional failure. Local control, disease-free survival and overall survival at 3 years were 98.9% (95%CI 92.2–99.8), 97.2% (95%CI 88.9–99.3), and 96.0% (95%CI 84.9–99.0), respectively. Five-year local control, disease-free survival, and overall survival rates were 96.3% (95%CI 84.7–99.2), 94.6% (95%CI 83.2–98.3), and 92.5% (95%CI 80.4–97.3), respectively. Patient self-reported cosmetic outcome was available for 51 patients, with all patients reporting being either very pleased, pleased, or satisfied with their cosmetic outcome, and no patients reported being dissatisfied or worse.

**Conclusions:**

The results of our series suggest the feasibility of utilizing IORT in a community-based cancer center with a high degree of local control, and patient satisfaction with regard to cosmesis. While the results of this series suggest that IORT may be a promising modality, longer follow-up is warranted to better understand exactly which clinicopathological features can predict long-term locoregional disease control.

## Introduction

A number of clinical trials, including the Early Breast Cancer Trialists’ Collaborative Group (EBCTCG) meta-analysis, support the use of adjuvant whole breast radiation therapy following the completion of breast conservation surgery to reduce locoregional risk of recurrence and reduce death from breast cancer [1]. As a result, adjuvant whole breast radiation therapy has developed into the standard of care for patients following a lumpectomy for early-stage breast cancer. Receiving daily radiation therapy, however, can present a logistical problem for some patients. In these cases, patients sometimes opt to undergo mastectomy or forgo radiotherapy altogether in order to avoid the complexities associated with external beam radiotherapy [2, 3]. To address these concerns, accelerated partial breast radiation therapy (APBI) and intraoperative radiation therapy (IORT) have been developed, which aim to reduce treatment time as well as volume of breast tissue irradiated. By placing the radiation source directly within the tumor bed, the utilization of IORT has the potential to substantially decrease the amount of normal tissue irradiated, thereby potentially enhancing the therapeutic ratio [4].

In 2014, Vaidya et al. [5] reported 5-year results of the TARGIT trial, a randomized, multi-institutional clinical trial comparing standard whole breast radiotherapy and intraoperative radiation therapy in patients with invasive breast cancer delivered with 50 kV photons. These results showed high rates of local control following a single fraction of IORT given at the time of surgery. At the same time, this trial showed slightly inferior rates of local control compared to the standard arm, not seen in other partial breast accelerated techniques [6, 7]. As a result, there has been ongoing considerable debate regarding the use and role of IORT, particularly due to the limited data and follow-up [8, 9].

In this series, we report our single institutional experience at a community-based cancer center using IORT as part of planned adjuvant monotherapy for patients with early-stage breast cancer.

## Materials and methods

Patients treated at a community-based hospital between 2011 and 2017 were retrospectively reviewed. All patients were evaluated pre-operatively in a multidisciplinary clinic setting, involving providers from radiology, pathology, breast surgery, medical oncology, and radiation oncology prior to confirming suitability for breast conservation therapy and intraoperative radiation therapy. Patients are considered candidates for IORT if they have clinical T1N0 disease. Although patients with tumors > 3 cm were generally excluded from IORT treatment, one patient with a clinically small tumor was found to have a final tumor size of 4.4 cm. Patients with known multi-focal, multi-centric disease, or disease not amenable to breast conservation surgery were excluded. Generally speaking, patients with estrogen negative disease were not considered for IORT, but some selected patients were nevertheless offered IORT depending on specific clinical situations. Compliance with anti-estrogen therapy in estrogen receptor-positive patients was strongly encouraged following the completion of surgery and IORT. Patients with tumors close to the skin were also excluded, and a post-lumpectomy skin bridge thickness of at least 1 cm was required to prevent excessive radiotherapy dose to the skin. All patients had confirmation of a negative margin of resection on intraoperative specimen radiography and no evidence of sentinel lymph node involvement on frozen section or touch prep. The details of the IORT procedure are similar to that of electronic brachytherapy techniques described by Vaidya et al. [10] and have been described elsewhere [11]. Patients were treated intraoperatively at the time of lumpectomy with 50 kV photons using the Xoft© electronic brachytherapy system to a dose of 2000 cGy, prescribed to the surface of spherical applicator balloons with patient-specific liquid water fill volumes. Demographic, clinical, radiographic, pathologic, and treatment outcomes were captured. Patients were followed with clinical breast examinations on at least a 6-month basis and mammography on a 6-month to 1 year interval as determined by radiology. Any ipsilateral breast failure was considered a local failure for the purposes of this analysis. Permission was obtained from the Institutional Review Board to complete this retrospective study.

## Results

Demographic information for patients in this series is depicted in Table 1. A total of 110 intraoperative radiation administrations were performed in 109 patients (one patient was treated for bilateral breast cancer). For the purposes of this analysis, each administration was analyzed as a separate event. The median tumor size was 9.3 mm, range (1–44 mm). The majority (69.7%) of patients had a diagnosis of invasive ductal carcinoma, and 27.5% of patients had ductal carcinoma in situ (Table 1).

**Table 1:** Demographic information

Total number of patients	109
Total number of intraoperative radiation treatments	110
Median age (years)	67, range (46–86 years)
Median tumor size (mm)	9.3, range (1–44 mm)
Tumor histology	
Invasive ductal carcinoma	76 (69.1%)
Ductal carcinoma in situ	30 (27.3%)
Invasive lobular carcinoma	3 (2.7%)
Metaplastic carcinoma	1 (0.9%)
Estrogen receptor status	
ER+	106/110 (96.4%)
ER−	4/110 (3.6%)
PR+	95/110 (86.4%)
PR−	15/110 (13.6%)
ER+/PR+	94/110 (85.4%)
ER+/PR−	11/110 (10.0%)
ER−/PR+	0 (0%)
ER−/PR−	4 (3.6%)
ER+/PR not reported	1 (0.9%)

**Fig. 1:**
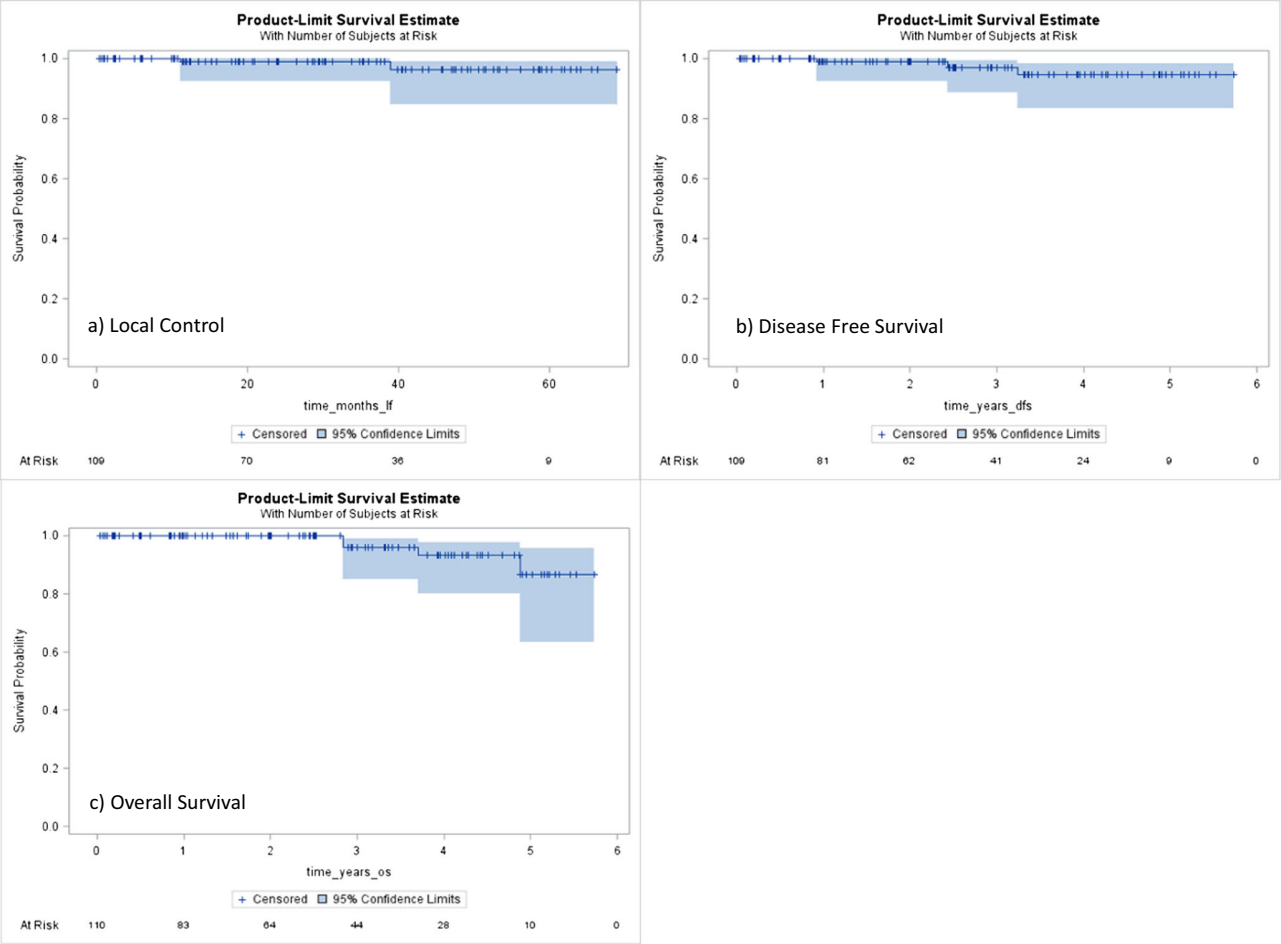
**a**–**c** Local control, disease-free survival, and overall survival. **a** Local control at 3 years was LC: 98.9% (95%CI 92.2–99.8). Local control at 5 years was 96.3% (95%CI 84.7–99.2). **b** Disease-free survival at 3 years was: 97.2% (95%CI 88.9–99.3). Disease-free survival at 5 years 94.6% (95%CI 83.2–98.3). **c** Overall survival at 3 years was 96.0% (95%CI 84.9–99.0). Overall survival at 5 years, 86.5% (95%CI 63.3–95.5)

**Fig. 2:**
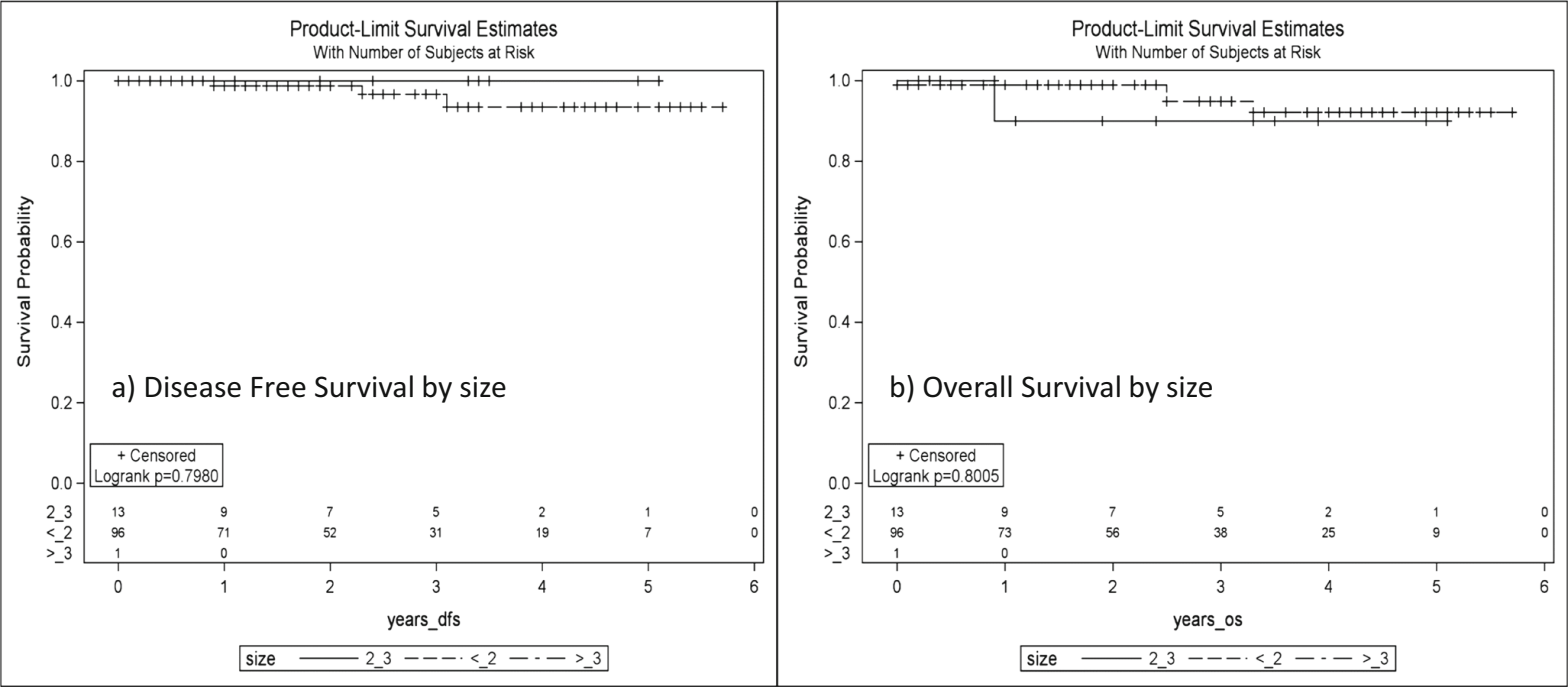
**a**–**b** Disease-free survival and overall survival by size. No significant differences were noted with regard to disease-free survival and overall survival as stratified by tumor size

Most of the patients in this series had estrogen positive disease (Table 1), and as a result, patients were generally offered anti-estrogen therapy. A total of 82 patients (75.2%) accepted anti-estrogen therapy, 20 (18.3%) patients either declined or did not tolerate this therapy (Table 2). Local control, disease-free survival, and overall survival at 3 years were 98.9% (95%CI 92.2–99.8), 97.2% (95%CI 88.9–99.3), 96.0% (95%CI 84.9–99.0), respectively. Five-year local control, disease-free survival, and overall survival rates were 96.3% (95%CI 84.7–99.2), 94.6% (95%CI 83.2–98.3), and 92.5% (95%CI 80.4–97.3), respectively (Fig. 1a–c). There were not any significant differences in disease-free survival and overall survival as stratified by size (Fig. 2a, b). Size as analyzed as a continue value (cm) had an overall survival HR 1.92 (95%CI 0.71–5.17) *p* = 0.199. Size as analyzed as continue value (cm) had a disease-free survival HR 0.70 (95%CI 0.11–4.38) *p* = 0.703.

**Table 2:** Additional therapy

Anti-estrogen therapy (*n* = 109)	
Anastrazole	56 (51.3%)
Tamoxifen	20 (18.3%)
Letrozole	3 (2.8%)
Combination tamoxifen/aromatase inhibitor	3 (2.8%)
Total anti-estrogen therapy	82 (75.2%)
Patient declined or did not tolerate	20 (18.3%)
Estrogen negative disease	4 (3.7%)
Missing	3 (2.8%)
Chemotherapy	7/109 (6.4%)
Re-excision	3 (2.7%)
Whole breast radiation therapy	12 (11.0%)
Reasons for whole breast radiation therapy	
Positive/close margin	4 (3.6%)
Positive lymph node	5 (4.5%)
Multi-focal disease	2 (1.8%)
Unable to locate lymph node	1 (0.9%)
Large tumor size on final path (> 4 cm)	1 (0.9%)
Additional Surgery	6 (9.6%)
Mastectomy	3 (2.7%)
Persistently positive margins	1 (0.9%)
Local recurrence	1 (0.9%)
Residual DCIS	1 (0.9%)
Local re-excision for positive margin	3 (2.7%)

Twelve patients (11.0%) required adjuvant whole breast radiotherapy for either close or positive margins, positive lymph nodes, multi-focal disease, or the inability to locate a lymph node (Table 2). Six patients required additional surgery, either with a mastectomy (3 patients, 2.7%) or margin re-excision (3 patients, 2.7%). These patients were included in this analysis of patients treated with IORT as part of planned monotherapy.

At a median follow-up of 29.9 months, two patients (1.8%) experienced a local failure in the ipsilateral breast; one patient was diagnosed with DCIS and the other patient developed recurrent invasive disease. Additionally, one patient (0.9%) experienced a regional nodal failure. Two patients (1.8%) experienced a contralateral breast failure. Both patients with local failures did not take anti-estrogen therapy (Table 3). Patient self-reported cosmetic outcomes were available for 51 patients, with all patients reporting being very pleased, pleased, or satisfied with their cosmetic outcome (Table 4).

**Table 3:** Disease control

Number of local failures	2 (1.8%)
Ipsilateral regional failure	1 (0.9%)
Time to locoregional failure	
Patient 1 (local failure)	38 months
Patient 2 (local failure)	11 months
Patient 3 (regional failure)	28 months

**Table 4:** Patient self-reported cosmesis (*n* = 51)

Very pleased	47 (92.1%)
Pleased	3 (5.9%)
Satisfied	1 (2.0%)

There did not appear to be any definitive association with age and use of IORT. The age adjusted HR for local control was 0.98 (95%CI 0.86–1.12), *p* = 0.777, and the age adjusted HR for disease-free survival was 0.99 (95%CI 0.88–1.11), *p* = 0.887.

## Discussion

The results of our series, with 5-year local control of 96.3% and a 5-year disease-free survival of 94.6% are consistent with other reports showing high rates of local control following a single fraction of IORT for patients with early-stage breast cancer [10, 12]. In addition to acceptable rates of local control, our series reported a high level of patient satisfaction with regard to cosmetic outcome, suggesting it is an appropriate treatment option for well-selected patients. Due to the small number of local failures in our series, it is not possible to perform a detailed analysis regarding factors that could predict for a local recurrence. We did not note any significant differences in disease-free survival when stratified by tumor size. While the two patients with local failures did not take anti-estrogen therapy, the numbers are too small to draw any definitive conclusions regarding the benefits of anti-estrogen therapy in a population of patients receiving IORT.

Patients were selected to receive IORT based on a discussion in a multidisciplinary setting, including breast surgery, radiation oncology, medical oncology, radiology, and pathology. We believe that this multidisciplinary approach is an important component to our intraoperative radiation therapy program, since patients with pathologically and radiographically adverse features are often not suitable candidates for this therapy. Although our database did not capture tumor grade, patients with high-grade tumors or multifocal disease are often excluded.

According to recently published, American Society for Radiation Oncology guidelines, patients are considered “suitable” for partial breast irradiation if they are: older than the age of 50 and have negative margins after surgical resection. Patients with DCIS are considered suitable if patients have a screen detected lesion, low to intermediate grade, lesion < 2.5 cm, and margins of resection that are negative at ≥ 3 mm [13]. However, the current ASTRO guidelines urge caution with regard to use of IORT using 50 kV photons except in the context of a clinical trial citing concerns of potential increases in local recurrence. In our series, over 25% of the patients had DCIS, providing at least some information that well-selected patients can safely receive IORT. Although this series was a retrospective review of patients treated at our institution, our current practice is to treat patients based on newly defined guidelines, and we offer eligible patient enrollment on a single-arm prospective clinical trial [14].

It is our experience that an IORT program may increase utilization rates of radiotherapy following a lumpectomy, and many patients who receive IORT may have forgone radiation therapy altogether. In addition to patient convenience, IORT may result in potential cost savings to the health care system and patient [15, 16]. As existing data becomes more mature, the actual benefit regarding cost-effectiveness may become better understood since one may be better able to account for all the costs associated with follow-up and subsequent treatment in a population of patients treated with IORT.

During pre-operative evaluation for patients with early-stage breast cancer, patients are presented treatment options considered appropriate with regard to the management of early-stage breast cancer. Based on the data from CALGB 9343 [17] and the PRIME II trial [18], some patients are offered surgery with anti-estrogen therapy, and no radiation therapy based on the concept that while radiotherapy provides a benefit in terms of a relative risk reduction, the absolute benefit is small. When selected patients are presented with the option of surgery with no further radiotherapy, surgery with a single fraction of IORT, or surgery followed by 3–5 weeks of post-operative external beam radiation therapy, often surgery with IORT is an appealing option. We believe that it is important to present patients with the rationale behind these options, and cite the data behind each strategy until more robust information exists and longer follow-up in patients who were treated with IORT is available.

We believe there are limitations of both the IORT procedure and our study that warrant further discussion. While intraoperative radiation therapy offers a convenient, focused alternative option compared to standard whole breast radiation therapy, we believe that there are noteworthy points that are necessary to discuss with patients prior to the administration of IORT. Unlike other forms of accelerated partial breast irradiation, when patients are treated with IORT, the final pathological details are not known. It is important to discuss these nuances with the patient, and how these unknown variables may impact a patient’s overall treatment plan. While our practice is to confirm negative lymph nodes on frozen section or touch prep and negative margins based on intraoperative specimen radiograph, some patients are found to have node-positive disease or positive margins despite initial indications that these were negative. Often these patients will go on to have re-excision surgery and/or external beam radiation therapy as clinically appropriate. It is our practice at the time of initial consultation discuss the indications for which additional treatment may be recommended, as in our series over 10% of patients went on to have adjuvant external beam radiation therapy, and nearly 10% of patients required additional surgery. Although the number of patients who received external beam radiation therapy is small, and the follow-up is still limited, we did not notice any significant toxicities in patients who went on to receive adjuvant whole breast radiation therapy. Our experience is consistent with the published data using IORT as a boost [19]. While the decision regarding adjuvant external beam radiation therapy is individualized, our practice is to offer external beam radiation treatment in node-positive patients, pending the final results of NSABP-B39 which included patients treated with partial breast irradiation therapy who were node positive [20].

While our early experience with IORT suggests favorable local disease control, it is important to exercise caution with regard to the utilization of IORT, as our long-term follow-up is only approximately 30 months. Our ability to draw conclusions with regard to 3- and 5-year outcomes is limited, and these limitations are reflected in the relatively large confidence intervals. Due to the patients who had to be censored due to lack of follow-up, the 95% confidence interval for 5 year-local control was between 84.7 and 99.2. Another limitation of this study is that while we have limited data with regard to physician reported cosmesis and side effects. However, our clinical experience is consistent with the favorable patient reported outcomes, with no patients reporting being dissatisfied with outcome. Our clinical experience is that patients have a side effect profile similar to that or better than standard whole breast radiation therapy. We are not aware of any grade 3 or higher acute or late skin toxicity based on our follow-up experience. Certainly critical to the avoidance of skin toxicity is only offering this modality to patients who have a favorable tumor location.

In conclusion, our series suggests the feasibility of utilizing IORT in a community-based cancer center with a high degree of patient satisfaction on the basis of cosmesis as well as a high level of local control. While it appears that patients with small, low grade, estrogen positive tumors would be good candidates for IORT, given the limited follow-up, longer follow-up data of existing study populations would help to better understand exactly which clinicopathological features are suitable for IORT. Additionally, we believe it is important to counsel patients that the randomized data [10] suggests that IORT may have a slightly higher rate of local recurrence compared to standard whole breast irradiation. Pending the maturation of more robust clinical trial information, we agree that IORT should be implemented in accordance with current ASTRO guidelines with regard to partial breast irradiation, and we agree with the recommendation that appropriate patients be enrolled on a clinical trial.
